# Development of a Three-Dimensionally Printed Ultrasound-Guided Peripheral Intravenous Catheter Phantom

**DOI:** 10.7759/cureus.17139

**Published:** 2021-08-13

**Authors:** Ting Xu Tan, Ying Ying Wu, Ian Riley, Youyou Duanmu, Samuel Rylowicz, Kenji Shimada

**Affiliations:** 1 Department of Emergency Medicine, Stanford University School of Medicine, Stanford, USA; 2 Department of Biomedical Engineering, Carnegie Mellon University, Pittsburgh, USA; 3 Department of Mechanical Engineering, Carnegie Mellon University, Pittsburgh, USA

**Keywords:** ultrasound-guided, simulation trainer, simulation in medical education, peripheral vascular, teaching procedures, emergency medicine procedures, three-dimensional (3d) printing, low-cost task trainers

## Abstract

Introduction

Ultrasound-guided peripheral intravenous catheter (US-PIVC) placement is an effective technique to establish PIV access when the traditional approach fails. Many training programs utilize commercial or homemade phantoms for procedural training. However, commercial products tend to be expensive and lack realism, while homemade blocks tend to be single-use and degrade easily. Thanks to the increasing availability of three-dimensional (3D) printers in academic settings, we sought to design and develop a reusable 3D-printed US-PIVC phantom and to evaluate its utility in terms of time needed to achieve IV placement and perceived realism compared to a commercial model among a group of emergency medicine (EM) physicians.

Methods

The upper extremity vascular phantom was constructed using 3D printing and casting techniques. A convenience sampling of EM physicians was timed by placing a US-PIVC in the 3D-printed and commercial models. Participants were also surveyed to assess their impression of the realism of the models. The primary outcome was the time required for US-PIVC placement in the 3D-printed model compared to the commercial model. Secondary outcomes were the assessment of differences in perceived realism and total cost between the two models.

Results

Twenty-one EM physicians completed the study. There were no significant differences in the mean time (seconds) for US-PIVC placement in the 3D-printed model (31, SD: 21) compared to the commercial model (30, SD: 18), p=0.77. Mean realism score trended higher for the 3D-printed model (3.6, SD: 0.9) compared to the commercial model (3.1, SD: 1.0), p=0.10. The total cost for the 3D-printed model was $120, with the interchangeable replacement part costing $21, which was much cheaper compared to the commercial phantom, which cost $549.

Conclusion

We developed a 3D-printed reusable US-PIVC phantom, and it proved to be more economical without sacrificing the realism and time required for US-PIVC placement when compared to a commercial phantom.

## Introduction

Peripheral vascular access is one of the most common procedures in medicine with 150-200 million peripheral intravenous catheters (PIVCs) placed in North America annually [1]. In the emergency department (ED), PIV access is essential for patient care and departmental workflow due to the time-sensitive need to administer fluids, intravenous contrast for imaging and medications, and to obtain laboratory specimens. In patients where the traditional approach of vein visualization and palpation is difficult to obtain, ultrasound guidance has been shown to increase success rates of peripheral venous access, improve patient satisfaction, and is associated with reductions in the number of central venous catheter placements [2-4].

While placing an ultrasound-guided peripheral intravenous catheter (US-PIVC) is an essential technique for emergency providers, there is no standardized method for teaching the procedure to trainees. Many training programs utilize either homemade blocks or commercially available task trainers in the form of rectangular blocks containing several vessels traversing the block. These models may not mimic the contours of the upper arm or the complex vessel anatomies found in patients. Commercial products also tend to be expensive and lack customizability, while traditional homemade blocks made of materials such as animal models, gelatin or, agar tend to degrade easily or require special storage conditions [5,6]. 

The use of three-dimensional (3D) printing in procedural teaching has expanded in recent decades and has been utilized across various specialties such as surgery, anesthesia, emergency medicine (EM), and obstetrics and gynecology [7-10]. Thanks to the increasing availability of 3D printers in academic settings, we sought to design and develop an economical, reusable, and customizable 3D-printed US-PIVC phantom that can be used as an initial prototype for building US-PIVC trainers with more varied and complex vessel anatomies in the future. We then evaluated this prototype’s utility regarding the time needed to achieve US-PIVC placement and perceived realism compared to a commercial model among a group of EM physicians.

This article was previously presented as a lightning oral presentation at the 2021 SAEM Annual Conference Meeting on May 12, 2021.

## Materials and methods

Phantom development

**Figure 1:**
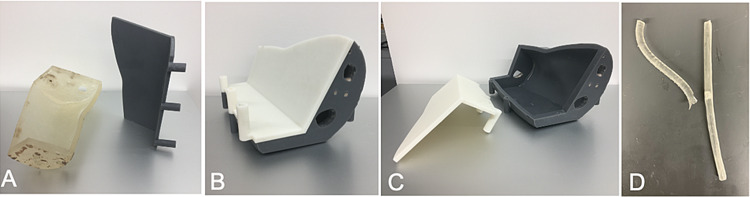
Molds for casting ballistic gels for the US-PIVC phantom A) Inner arm mold. B) Outer arm mold. C) Removable portion of outer arm mold. D) Vessel mold and an example of a vessel bifurcation US-PIVC: ultrasound-guided peripheral intravenous catheter

The phantom developed was constructed using 3D printing and casting techniques. It consisted of three parts: an outer arm base with a humerus bone, an interchangeable inner arm containing vessels, and a removable skin layer. The molds for the arms, bone, and vessels were developed from a CAD model of an anatomical arm, which was scaled and customized to create the target training case using an open-source mesh editing software Blender (Blender 2.78, Blender Foundation). The molds and humerus bone were then 3D-printed (Figure 1) using the Form 2 stereolithography (SLA) 3D printer (Formlabs, Somerville, MA) and its proprietary photopolymer resin.

The upper arm base was a separate piece from the inner arm as the inner arm was expected to wear out faster. This arrangement would reduce the cost of replacement when maximum durability was reached or if a different type of vessel diameter or trajectory was desired. The inner arm contained three vessels: the basilic vein, brachial artery, and cephalic vein. To decrease air artifact around the vessels during molding, silicone tubing was not used to model the vessel walls. Instead, vessels were modeled as negative space left behind by vessel molds that were removed once the inner arm material had set in.

**Figure 2:**
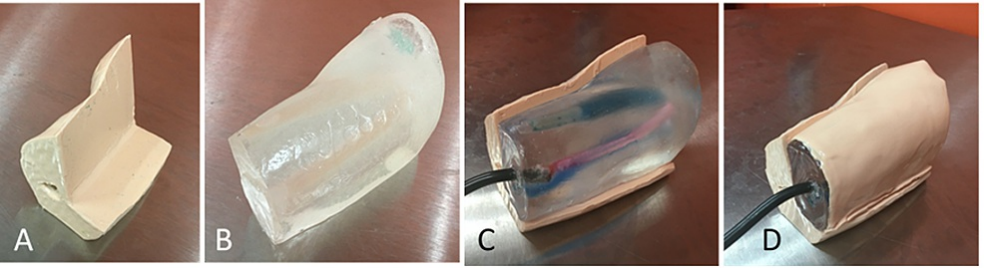
Components of the initial prototype A) Outer arm base with humerus bone. B) Inner arm. C) Assembled outer and inner arm. D) Assembled outer and inner arm with silicone skin over the inner arm

For the initial prototype (Figure 2), pure 10% FBI-quality ballistic gelatin (Clear Ballistics, Greenville, SC) was used to cast the inner arm with a vessel diameter of 1 cm (Figure 2B). A hand pump from a manual blood pressure cuff was attached to the end of the brachial artery and sealed using ballistic gel to enable the user to simulate pulsations in the brachial artery. The outer arm base was made of Dragon Skin 10NV two-part silicone (Smooth-On, Inc, Macungie, PA) with a slacker (Smooth-On, Inc) added in a 1:1:1.5 ratio to reduce the shore hardness of silicone. Pigments were added to the silicone by mixing white and flesh tone pigments (Silc Pig, Smooth-On, Inc) in a 1:1 ratio to generate the skin tone shown in Figure 2A. The humerus bone was inserted into the silicone in the outer arm to add more anatomical realism. A removable skin layer to cover the inner arm was made from Dragon Skin 10 Fast silicone (Smooth-On, Inc) and the same pigments. To create a realistic skin layer, uncured silicone was allowed to spread over the ballistic gel and cure, so that the skin layer followed the ballistic gel’s contours. Figure 2D shows the assembled US-PIVC phantom.

**Figure 3:**
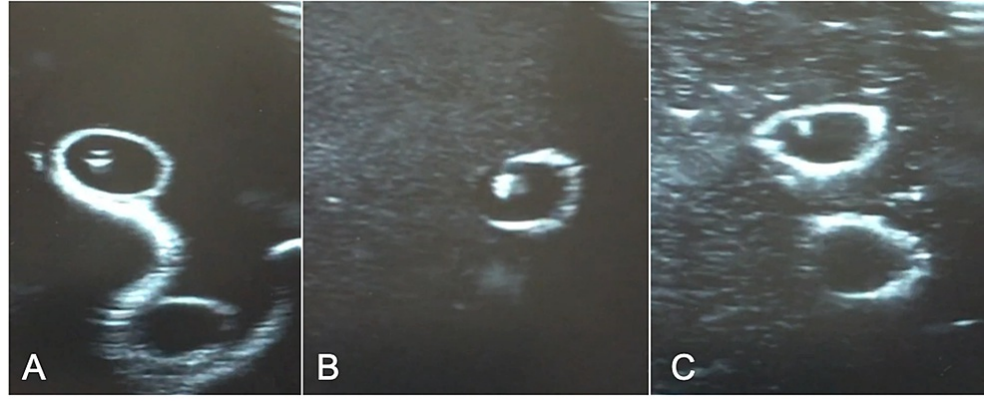
Echogenicity of models under ultrasound with the needle in the vessel A) Pure 10% ballistic gel. B) Corn starch and ballistic gel. C) Chalk and ballistic gel

In subsequent iterations of the model, the vessel diameter was decreased to 0.5 cm to increase the model’s difficulty level. Additives such as corn starch (Argo, Oakbrook Terrace, IL) and chalk powder calcium carbonate (LD Carlson, Kent, OH) was added to the ballistic gel to increase the echogenicity of the model under ultrasound (Figure 3). The ratio of each additive to ballistic gel was 0.2 g of corn starch to 100 ml ballistic gel and 0.1 g chalk to 100 ml ballistic gel.

Prototype evaluation

A randomized, non-blinded educational study was performed to assess the performance of the prototype. The study population consisted of a convenience sampling of EM physicians at an academic tertiary center. Each participant was randomized using an Excel random number generator (Microsoft, Seattle, WA) to first place a US-PIVC in either the 3D-printed model or the commercial model (Blue Phantom, CAE Health Care, Seattle, WA), and then to subsequently place a US-PIVC in the other model. The time required from the start of the procedure to successful vein cannulation for each participant on each model was recorded by a study investigator. Participants were surveyed after completing the task to assess the realism of each model compared to US-PIVC placement in a real patient on a five-point Likert scale, with five indicating “very realistic” and one indicating “very unrealistic”. The primary outcome was the time required for successful US-PIVC placement in the 3D-printed model compared to the commercial model. Secondary outcomes were differences in perceived realism and total cost between the two models. Two-tailed t-tests were used to compare IV placement times and realism scores. The US-PIVC placement times and realism scores were reported as mean with standard deviation. Two-tailed t-tests were used to compare the data points.

## Results

A total of 21 participants [eight attendings and 13 post-graduate year (PGY) 2-4 residents] participated in the study (Table 1). All study participants had prior experience in placing US-PIVCs in at least one patient and the majority (17, 81%) had prior formal US-PIVC training in the form of a simulation session.

**Table 1:** Participant characteristics PGY: post-graduate year; US-PIVC: ultrasound-guided peripheral intravenous catheter

Characteristics	N (%), (n=21)
Level of training	
PGY2	9 (42.9)
PGY3	2 (9.5)
PGY4	2 (9.5)
Attending	8 (38.1)
Prior formal US-PIVC training	
Yes	17 (81.0)
No	4 (19.0)
Participants starting with	
Commercial phantom	11 (52.4)
3D-printed phantom	10 (47.6)

For the primary outcome, there was no significant difference in the mean time (in seconds) for successful US-PIVC placement in the 3D-printed model (31, SD: 21) compared to the commercial model (30, SD: 18), p=0.77. There was also no significant difference in the mean time for the successful US-PIVC placement in the two models between the groups that were randomized to 3D-printed vs. commercial model first (Table 2). 

**Table 2:** Mean times in seconds for US-PIVC placement US-PIVC: ultrasound-guided peripheral intravenous catheter

	3D-printed phantom	Commercial phantom	P-value
Overall mean time in seconds (standard deviation)	31 (21)	30 (18)	0.77
Mean times in seconds for participants using 3D-printed phantom first (standard deviation)	36 (20)	29 (15)	0.39
Mean times in seconds for participants using commercial phantom first (standard deviation)	28 (23)	31 (20)	0.75

There was a trend towards a higher mean realism score for the 3D-printed model (3.6, SD: 0.9) compared to the commercial model (3.1, SD: 1.0) though this was not statistically significant (p=0.10). There was also a trend towards higher perceived usefulness of the model for teaching US-PIVC placement for the 3D-printed model (4.3, SD: 0.5) compared to the commercial model (4.1, SD: 0.6).

Cost

The total materials cost for the initial 3D-printed prototype was $120. This phantom was five times cheaper than the commercial phantom, which was $549 for the Blue Phantom Branched 4 Vessel Ultrasound Training Block Model (CAE Health Care) [11]. The material cost excluding the cost of the molds was $70. The material cost of the inner arm was only $21. The price breakdown for each material is summarized in Table 3.

**Table 3:** Breakdown of material costs

Part	Material	Cost
Outer arm base	Dragon Skin 10NV silicone	$25
	Slacker	$19
	Photopolymer resin	$5
Inner arm	Ballistic gel	$19
	Dragon Skin 10 Fast silicone	$2
Molds	Photopolymer resin	$50
Total	-	$120

## Discussion

We developed a 3D-printed US-PIVC phantom that was comparable in terms of time to placing a US-PIVC to the more costly commercial product without sacrificing realism. Time to place US-PIVC was chosen as the primary objective because our goal for this initial prototype was to adequately simulate the procedure as compared to the Blue Phantom model, which was the standard educational tool at our teaching institution, while still emphasizing participant success at IV insertion. Since our initial basic prototype was meant to be comparable to the Blue Phantom model, our hypothesis was that the timing to place US-PIVC between the two models should not be significantly different. Once the molds and arm parts were made, only the inner arm piece needed to be replaced should the user desire a different vessel layout or if degradation from repeated use occurred. Furthermore, the ballistic gel could be deconstructed and reused, thus minimizing additional costs to maintain the longevity of the 3D-printed US-PIVC model. Both silicone and ballistic gel also did not require special storage conditions, and previous models made by the authors using the same materials and specifications as this prototype have remained intact and usable for at least three years.

This study was also the first known study to compare a 3D-printed US-PIVC phantom to a commercial model in assessing time to IV placement and perceived realism in placing a US-PIVC in patients. There were two prior studies comparing the Blue Phantom model to a non-commercial task trainer: one a porcine model, another a non-3D-printed ballistics gel model [12,13]. Both studies, through survey data, showed that homemade models improved user confidence in the procedure. The porcine model fared similarly when compared to the Blue Phantom in terms of vessel compressibility, echogenicity, and vessel resemblance to humans under ultrasound [12]. The ballistics gel model, on the other hand, was perceived to be more realistic than the Blue Phantom with similar echogenicity to human anatomy [13]. Our study supports this body of work showing that non-commercial phantoms are comparable to the more expensive commercial models and adds to the current literature by providing objective procedure time comparisons.

This study had several limitations as it was a single-center study with small sample size. Furthermore, as a result of convenience sampling, all participants had prior experience placing a US-PIVC in patients, which may have affected the recorded time to US-PIVC placement. Since these phantoms are typically used by novice learners, including novices who have had no prior experience with US-PIVC placement could produce more interesting and conclusive results. The ability to make these phantoms are also limited by the training program’s access to 3D printers. Noticeable track marks within the ballistic gel were visible within a 1 cm^2^ area after more than 20 attempts. Signs of damage were apparent after 120 attempts. This is in comparison to the Blue Phantom model, which demonstrated “excellent durability after 1,000 needle punctures in a 1 cm^2^ area” [14]. The number of needle punctures for visible track marks was not commented on in the paper. There are also constraints in the curvature of the vessel and angle of bifurcation as the 3D-printed vessel molds are pulled out after the inner arm ballistic gel had set in.

Despite these limitations, the 3D-printed phantom prototype was found to be realistic, and the three-part assembly of the outer arm base, inner arm, and removable skin layer creates opportunities to reduce the difficulty of US-PIVC placement according to the trainee’s competency level. In particular, removing the skin enables the trainee to correlate the ultrasound images to the actual vessel positions in the model. Removing the skin also enables trainees to visualize their needle trajectory after each US-PIVC placement. The option to customize the model will also enable educators to generate models of varying difficulty in order to target trainees with varying levels of competency. While the ballistic gel is reusable, one aspect of improvement is the replacement of ballistic gel with a self-healing material to improve durability. The addition of corn starch and chalk can also improve the phantom by increasing the echogenicity of the ultrasound images and the difficulty in ultrasound guidance.

## Conclusions

We developed a novel 3D-printed US-PIVC model that was more economical without sacrificing the time required for IV placement or realism when compared to a commercial phantom. With the current availability of 3D printers, low cost of materials, and ease of storage, this model is an accessible task trainer for educational programs. The customizability of the phantom also encourages individualized training for different learner levels. Further studies will be needed to assess the clinical utility of this 3D-printed phantom prototype for medical education and to develop phantoms with more complex vessel anatomies.
